# Comprehensive analysis of immunogenic cell death-related gene and construction of prediction model based on WGCNA and multiple machine learning in severe COVID-19

**DOI:** 10.1038/s41598-024-59117-0

**Published:** 2024-04-11

**Authors:** Chunyu Li, Ke Wu, Rui Yang, Minghua Liao, Jun Li, Qian Zhu, Jiayi Zhang, Xianming Zhang

**Affiliations:** 1 Department of Respiratory and Critical Medicine, the Affiliated Hospital of Guizhou Medical University, Guiyang, 550004 Guizhou China; 2 School of Clinical Medicine, Guizhou Medical University, Guiyang, 550004 Guizhou China; 3 Department of Internal Medicine, Guiyang First People’s Hospital, Guiyang, 550004 Guizhou China

**Keywords:** COVID-19, Immunogenic cell death, Machine learning, Immune cell infiltration, Computational biology and bioinformatics, Diseases

## Abstract

The death of coronavirus disease 2019 (COVID-19) is primarily due to from critically ill patients, especially from ARDS complications caused by SARS-CoV-2. Therefore, it is essential to contribute an in-depth understanding of the pathogenesis of the disease and to identify biomarkers for predicting critically ill patients at the molecular level. Immunogenic cell death (ICD), as a specific variant of regulatory cell death driven by stress, can induce adaptive immune responses against cell death antigens in the host. Studies have confirmed that both innate and adaptive immune pathways are involved in the pathogenesis of SARS-CoV-2 infection. However, the role of ICD in the pathogenesis of severe COVID-19 has rarely been explored. In this study, we systematically evaluated the role of ICD-related genes in COVID-19. We conducted consensus clustering, immune infiltration analysis, and functional enrichment analysis based on ICD differentially expressed genes. The results showed that immune infiltration characteristics were altered in severe and non-severe COVID-19. In addition, we used multiple machine learning methods to screen for five risk genes (KLF5, NSUN7, APH1B, GRB10 and CD4), which are used to predict COVID-19 severity. Finally, we constructed a nomogram to predict the risk of severe COVID-19 based on the classification and recognition model, and validated the model with external data sets. This study provides a valuable direction for the exploration of the pathogenesis and progress of COVID-19, and helps in the early identification of severe cases of COVID-19 to reduce mortality.

## Introduction

Since December 2019, the coronavirus disease 2019 (COVID-19), caused by sever acute respiratory syndrome coronavirus 2 (SARS-CoV-2), has led to a widespread epidemic and high mortality worldwide. As of December 20, 2023, COVID-19 has infected more than 772 million people and has led to more than 6.9 million deaths, according to World Health Organization report^1^. The death of COVID-19 is primarily due to from critically ill patients, especially from ARDS complications caused by SARS-CoV-2^2^. Therefore, early prediction and identification of severe cases and accurate treatment are extremely important to reduce the mortality rate of COVID-19.

Based on current evidences, risk factors for developing into critical ill COVID-19 include demographic factors, such as older age, male, and the presence of underlying conditions such as hypertension, cardiovascular disease, chronic obstructive pulmonary disease (COPD), chronic kidney disease, and tumors^3^. A study has also shown that the risk factors of critical illness are related to troponin levels, C-reactive protein, D-dimer and other biochemical indicators^4^. However, none of these risk factors accurately predicted the occurrence of severe illness owing to individual differences between hosts. The construction of predictive models with multiple indicators, especially multiple biomarkers combined, may be more valuable. For example, Chen et al. built random forest models for predicting severe COVID-19 based on clinical features and laboratory results, with prediction accuracy exceeding 90% and 95%, respectively^5^. Based on transcriptomic data, Li et al. used machine learning methods to identify multiple biomarkers for predicting COVID-19 severity^6^. These studies suggest that machine learning models combining multiple indicators can be used to predict the severity of COVID-19 and guide clinical treatment. Therefore, it is essential to contribute an in-depth understanding of the pathogenesis of the severe COVID-19 and to identify biomarkers for predicting critically ill patients at the molecular level.

Studies have shown that the lung is the most frequently injured and severely injured organ in severe COVID-19 patients, mainly manifested as diffuse alveolar injury, accompanied by a large number of immune cell infiltration and inflammatory factor storm^7,8^. The uncontrolled inflammatory response develops into systemic inflammatory response syndrome, which causes tissue damage to the lungs and extra-pulmonary organs, and eventually leads to multiple organ dysfunctions^9^. The destruction and death of alveolar epithelial cells and inflammatory cells might be the initiation of the diffuse alveolar injury and cytokine storm caused by SARS-CoV-2 infection, and is one of the mechanisms causing the imbalance between coagulation activation and fibrinolytic inhibition^10^. It has been reported that various types of cell death are involved in the progression of severe COVID-19, such as apoptosis, pyroptosis, NETosis and necroptosis, and the regulation of cell death may be a new therapeutic target^11^.

Immunogenic cell death (ICD), as a specific variant of regulatory cell death driven by stress, can induce adaptive immune responses against cell death antigens in the host^12^. Studies have shown that ICD is involved in the progression of many diseases, especially in tumors and infectious diseases^13^. When cells are stimulated to produce ICDs, dying cells produce new epitopes and release damage associated molecular patterns (DAMPs), which are recognized by pattern recognition receptors (PRRs) and presented to T cells, further activating the adaptive immune response^14^. Studies have confirmed that both innate and adaptive immune pathways are involved in the pathogenesis of SARS-CoV-2 infection^15–17^. In severe COVID-19 patients, multiple DAMPs, including HMGB1 and S100 proteins, are significantly increased due to the massive release of inflammatory mediators and the occurrence of cell death^18–20^. DAMPs may contribute to the progression of severe COVID-19 by driving the uncontrolled immune response associated with COVID-19^21^. Thus, ICD may play an important role in the occurrence and progression of severe COVID-19. However, little is still known about the role of ICD in the pathogenesis of severe COVID-19.

In this work, we systematically analyzed the role of ICD-related genes in COVID-19. The differential expression of ICD-related genes and immune infiltration in COVID-19 samples and healthy control samples, as well in ICU samples and Non-ICU samples were respectively explored. A prediction model for severe COVID-19 was constructed based on ICD-related genes via consensus cluster analysis, weighted gene co-expression network analysis and multiple machine learning algorithms, and an external dataset were adopted to verify the performance of the predictive model. This work may provide valuable insights into the pathogenesis, progression and immunotherapy of COVID-19, as well as aid in the early identification of severe COVID-19.

## Results

### Differential expression landscape of ICD-related genes

**Figure 1:**
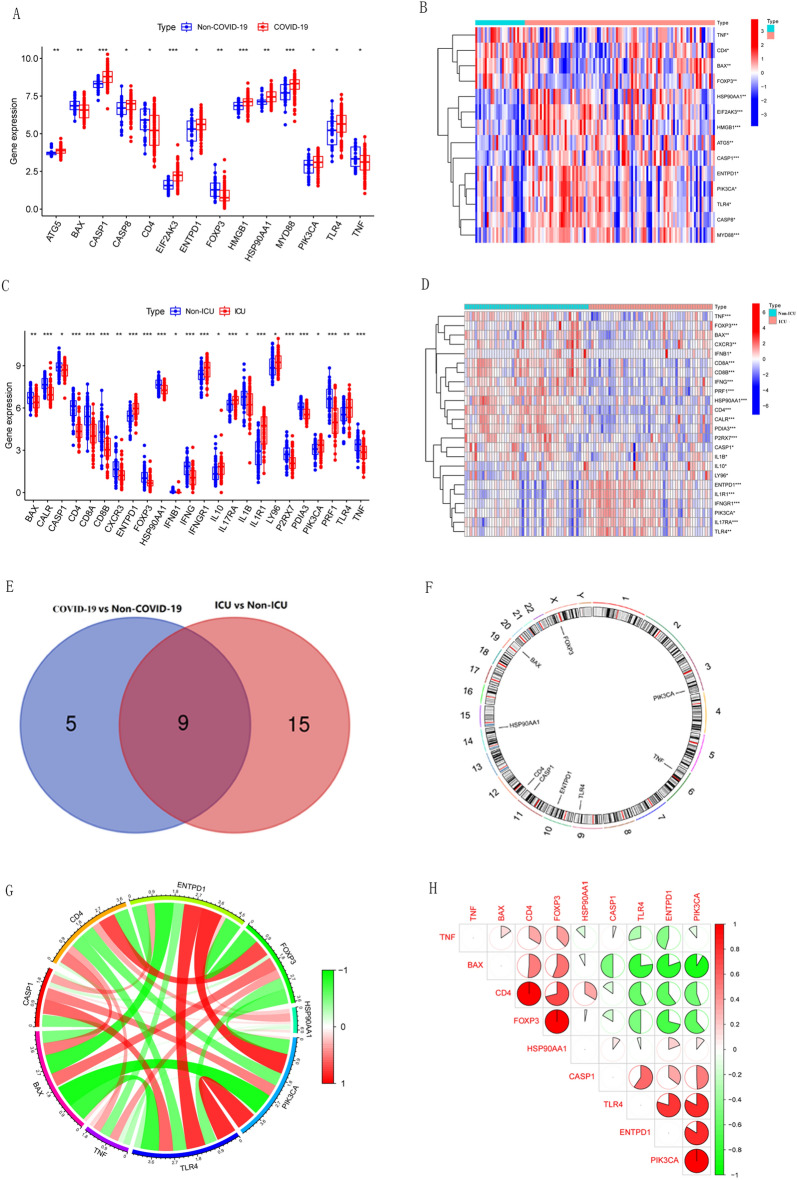
Differential expression landscape of ICD-related genes. (**A**,**B**) Heat map and boxplot of the expression of 14 ICD-related DEGs in COVID-19 and Non-COVID-19 samples. (**C**,**D**) Heat map and boxplot showed the expression of 8 up-regulated and 16 down-regulated ICD-related genes in ICU COVID-19 samples. (**E**) Venn diagram demonstrating Intersection of differentially expressed ICD-related genes in different subgroups. (**F**) The positions of the nine Intersection genes on the chromosomes. (**G**,**H**) The correlation circle plot and correlation heat map showed the relationship between the nine intersecting genes. Red and green represent positive and negative correlations, respectively. The correlation coefficient was displayed as the area of the pie chart. *p < 0.05, **p < 0.01, ***p < 0.001.

Based on the previously published literature^22^, we obtained 34 ICD-related genes. Differential expression of ICD-related genes was screened in COVID-19 samples and healthy control samples, as well in ICU samples and Non-ICU samples. The results showed that 10 ICD-related genes were up-regulated, including ATG5, CASP1, CASP8, EIF2AK3, ENTPD1, ENTPD1, HSP90AA1, MYD88, PIK3CA and TLR4, while 4 genes (BAX, CD4, FOXP3 and TNF) were down-regulated in the COVID-19 samples (Fig. 1A,B). Meanwhile, eight ICD-related genes were up-regulated and 16 genes were down-regulated in ICU COVID-19 patients (Fig. 1C,D). The two differentially expressed gene sets mentioned above were intersected to obtain DEGs associated with severe cases (i.e. ICU admission) in COVID-19 patients. A total of 9 differential genes (TLR4, HSP90AA1, PIK3CA, BAX, ENTPD1, CD4, CASP1, FOXP3, TNF) were obtained in the intersection of gene sets (Fig. 1E). At the same time, the positions of the nine differential genes on the chromosomes were visualized in Fig. 1F. Then, a correlation analysis of these genes was performed to explore the interactions between them (Fig. 1G,H).

### Immune infiltration and enrichment analysis in different severity groups

**Figure 2:**
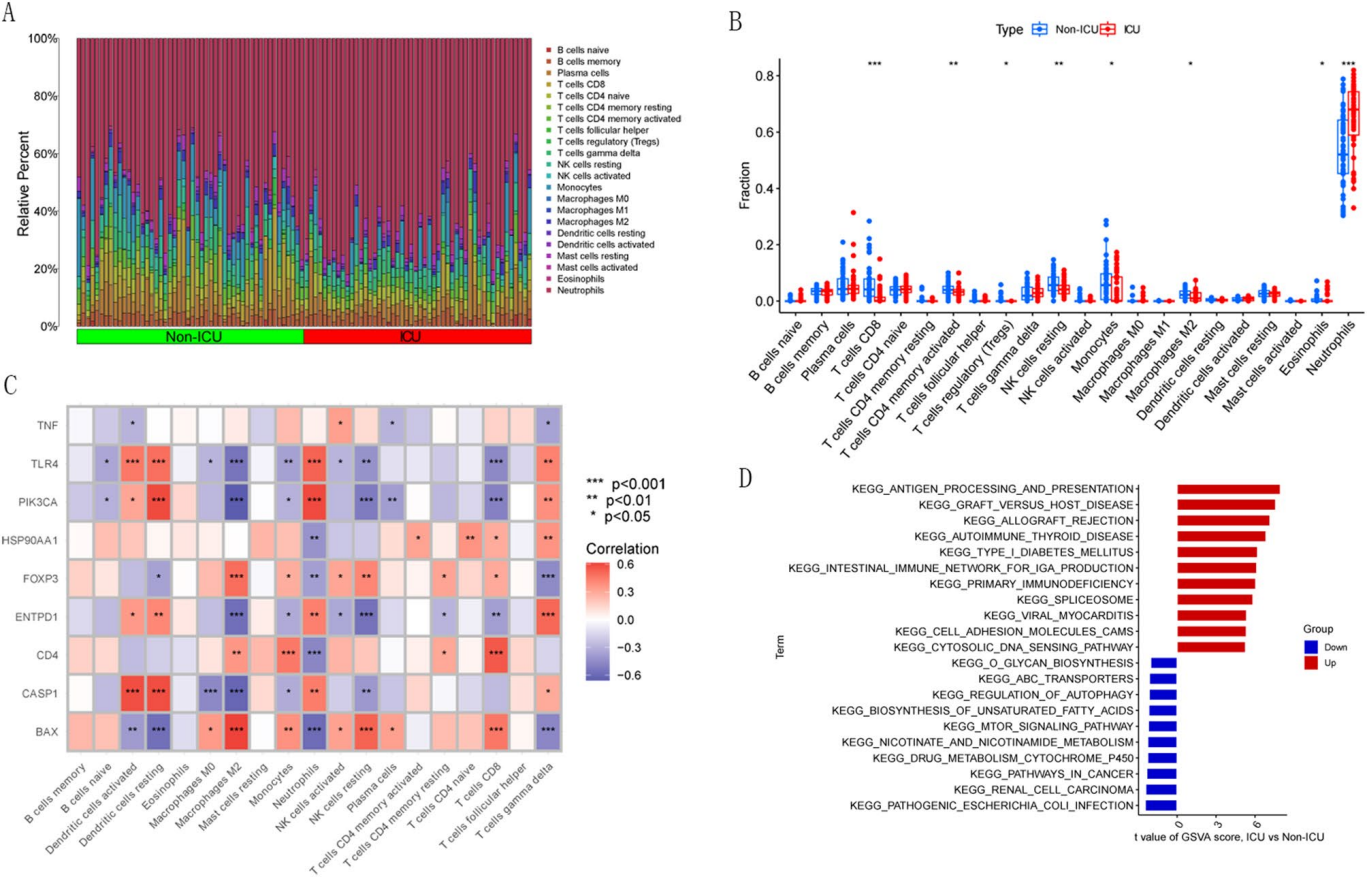
Immune infiltration and enrichment analysis in different severity groups. (**A**) Relative abundance of 22 infiltrating immune cells between ICU and Non-ICU COVID-19 samples. (**B**) Boxplot showed differences in immune infiltration between ICU and Non-ICU COVID-19 samples. (**C**) Heat map of correlation between 9 intersection genes and immune cell infiltration. (**D**) GSVA results of KEGG gene sets between ICU and Non-ICU COVID-19 samples were plotted in a bar plot. *p < 0.05, **p < 0.01, ***p < 0.001.

To explore the role of the immune system in critical COVID-19 cases, we performed an immune infiltration analysis between ICU and Non-ICU COVID-19 samples (Fig. 2A). The results demonstrated that ICU COVID-19 patients showed lower levels of infiltration in T cells CD8, T cells CD4 memory activated, T cells regulatory (Tregs), NK cells resting, Monocytes, Macrophages M2 and Eosinophils, Meanwhile, higher levels of infiltration in neutrophils (Fig. 2B). This suggests that immune cell infiltration plays an important role in the development of severe cases of COVID-19. Subsequently, we conducted immune infiltration analysis on nine differentially expressed intersection genes (Fig. 2C). The results showed that TLR4, PIK3CA, FOXP3, ENTPD1, CD4, CASP1, BAX were significantly correlated with a variety of immune cell infiltration (p < 0.001). It can be seen that ICD-related genes may play a key role in the regulation of severe COVID-19 and immune infiltration. Then, we performed GSVA based on Kyoto encyclopedia of genes and genomes (KEGG) gene sets. The KEGG results showed that antigen processing and presentation, primary immunodeficiency and viral myocarditis were upregulated in ICU COVID-19 group, while drug metabolism cytochrome P450 and MTOR signaling pathway were downregulated (Fig. 2D).

### Establishment of COVID-19 disease clusters based on ICD- related DEGs

**Figure 3:**
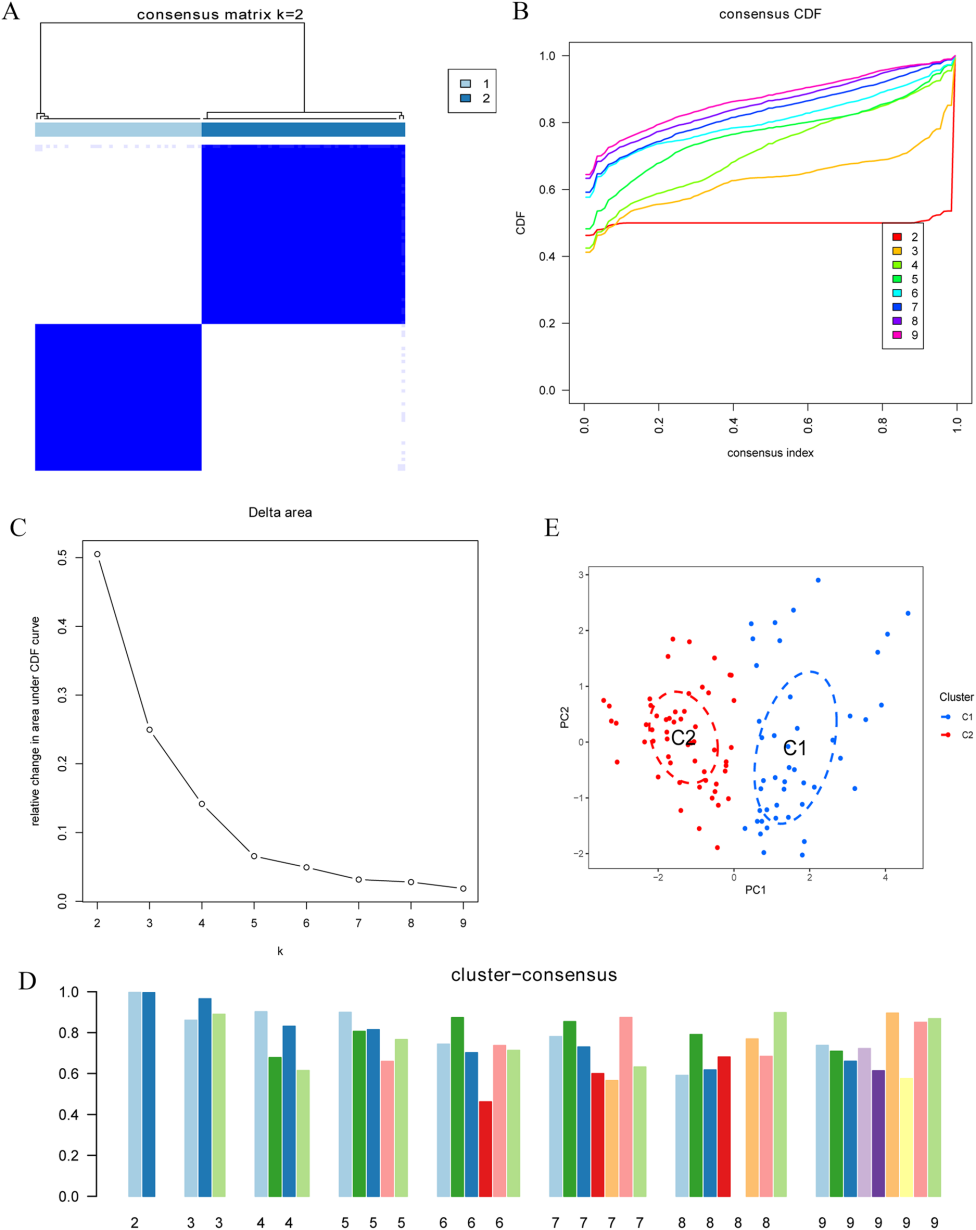
Establishment of COVID-19 disease clusters based on ICD- related DEGs. (**A**) Consensus clustering matrix when k is 2. (**B**) Representative cumulative distribution function (CDF) curve. (**C**) Representative CDF delta area curve. (**D**) Consensus clustering score when k is 2–9. (**E**) Visualization of the distribution of the two clusters by principal component analysis (PCA).

We used a consensus clustering algorithm to identify different groups of COVID-19 subtypes based on 14 ICD-related DEGs in COVID-19 samples and healthy control samples. The best consistent clustering was established when k = 2, with the least consensus index of the CDF curve and the relatively large consensus score (Fig. 3A–D). Thus, we divide COVID-19 samples into two clusters (C1 and C2). Besides, principal component analysis (PCA) showed that the COVID-19 samples can be distinguished between the two clusters based on the 14 ICD-related DEGs (Fig. 3E).

### Immune infiltration and enrichment analysis in different ICD clusters

**Figure 4:**
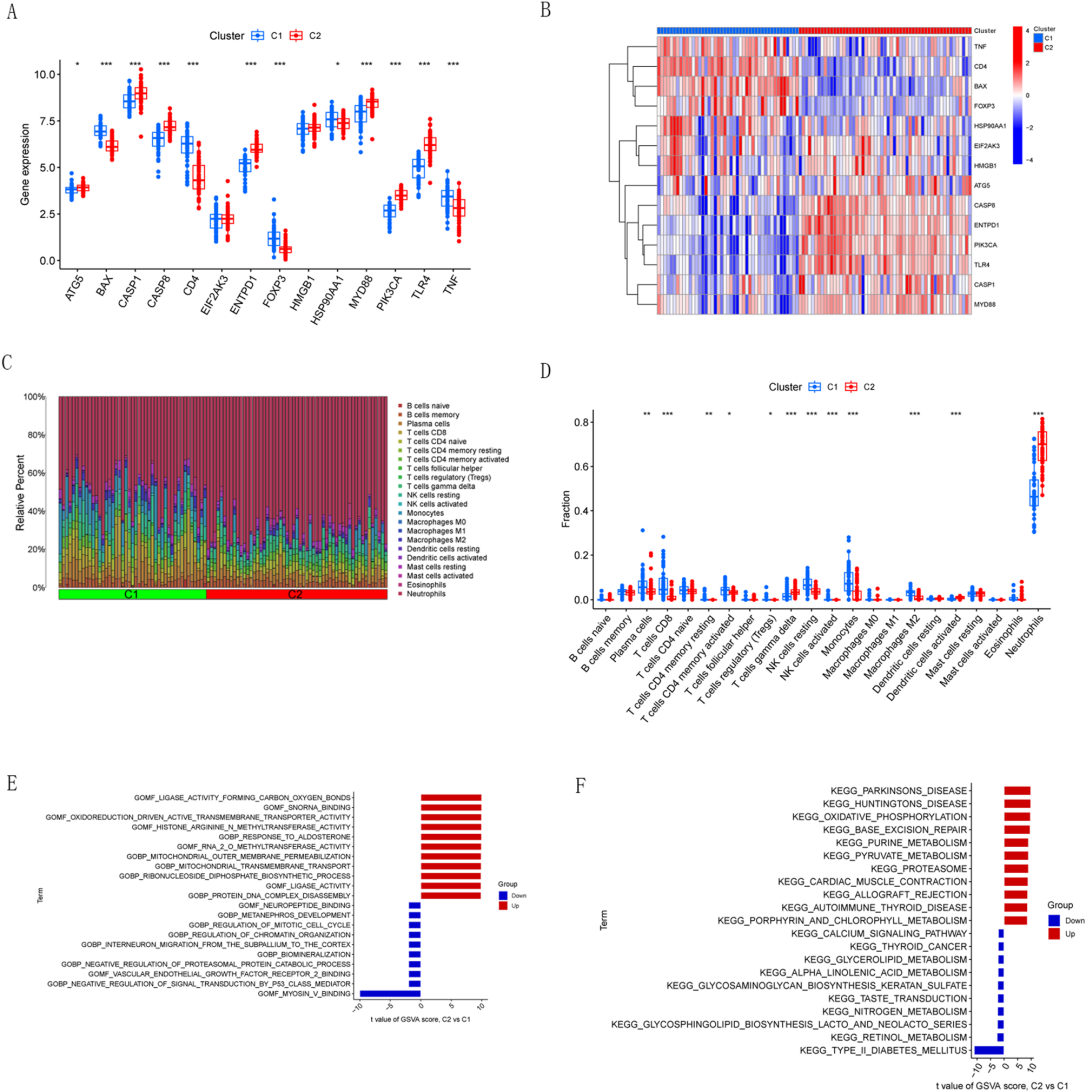
Immune infiltration and enrichment analysis in different ICD clusters. (**A**) Boxplot showed the differences in the expression of 14 ICD-related DEGs between the two clusters. (**B**) Heat map showed the expression profile of 14 ICD-related DEGs between two clusters. (**C**) Relative abundance of 22 infiltrating immune cells between the two clusters. (**D**) Boxplot showed the differences in immune infiltration between the two clusters. (**E**) GSVA results of GO gene sets between two clusters were plotted in a bar plot. (**F**) GSVA results of KEGG gene sets between two clusters were plotted in a bar plot. *p < 0.05, **p < 0.01, ***p < 0.001.

We analyzed the differential expression of the 14 ICD-related DEGs between the two ICD clusters and found that BAX, CD4, FOXP3, HSP90AA1 and TNF were upregulated in Cluster 1, while ATG5, CASP1, CASP8, ENTPD1, MYD88, PIK3CA, and TLR4 were upregulated in Cluster 2 (Fig. 4A,B). Immune infiltration analysis between the two ICD clusters showed that Cluster 2 had relatively lower infiltrating levels of Plasma cells, T cells CD8, T cells CD4 memory resting, T cells CD4 memory activated, T cells regulatory (Tregs), NK cells resting, NK cells activated, Monocytes and Macrophages M2 and relatively higher infiltrating levels of infiltrating and neutrophils (Fig. 4C,D). Then we performed GSVA based on gene ontology (GO) and KEGG gene sets. The GO results showed that response to aldosterone, ligase activity and protein DNA complex disassembly were upregulated in Cluster 2, while myosin V binding and vascular endothelial growth factor receptor 2 binding were downregulated in Cluster 2 (Fig. 4E). The KEGG results showed that Oxidative phosphorylation and base excision repair were upregulated in Cluster 2, while the calcium signaling pathway and type II diabetes mellitus were downregulated in Cluster 2 (Fig. 4F).

### Intersection gene screening based on gene modules of WGCNA

**Figure 5:**
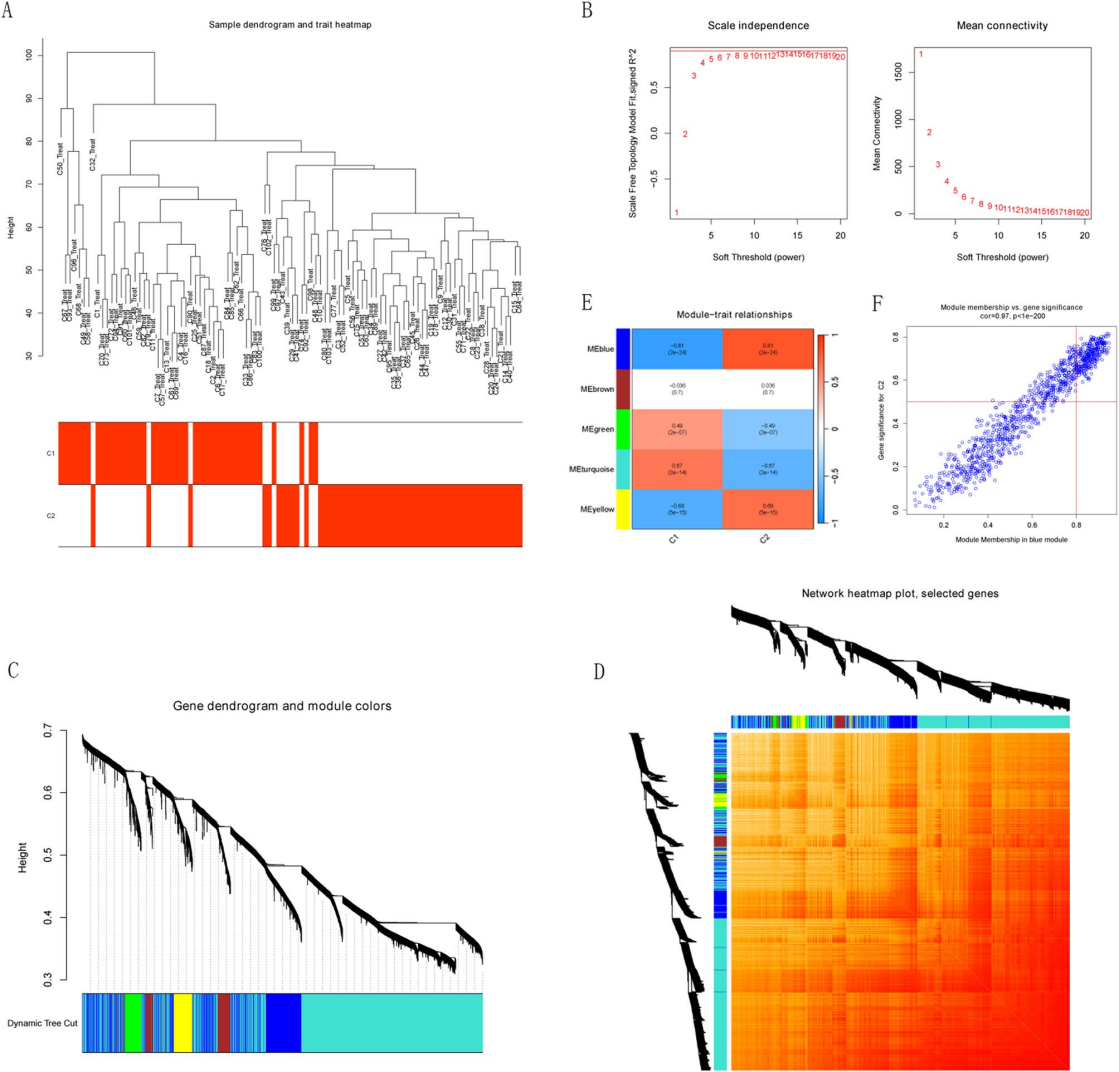
The WGCNA algorithm was used to identify key gene modules closely associated with the ICD cluster. (**A**) The sample dendrogram and feature heat map were drawn based on the Euclidean distance using the average clustering method for hierarchical clustering of samples, with each branch representing a sample, Height in the vertical coordinate being the clustering distance, and the horizontal coordinate being the ICD cluster information. (**B**) Soft threshold (power = 13) and scale-free topology fit index (R^2^ = 0.9). (**C**,**D**) Gene hierarchy tree-clustering diagram. The graph indicates different genes horizontally and the uncorrelatedness between genes vertically, the lower the branch, the less uncorrelated the genes within the branch, i.e., the stronger the correlation. (**E**) Heat map showed the relations between the Cluster 1 and Cluster 2. The values in the small cells of the graph represent the two-calculated correlation coefficients between the eigenvalues of each trait and each module as well as the corresponding statistically significant p-values. Color corresponds to the size of the correlation; the darker the red, the more positive the correlation; the darker the green, the more negative the correlation. (**F**) Scatter plot between gene salience (GS) and module members (MM) in blue.

**Figure 6:**
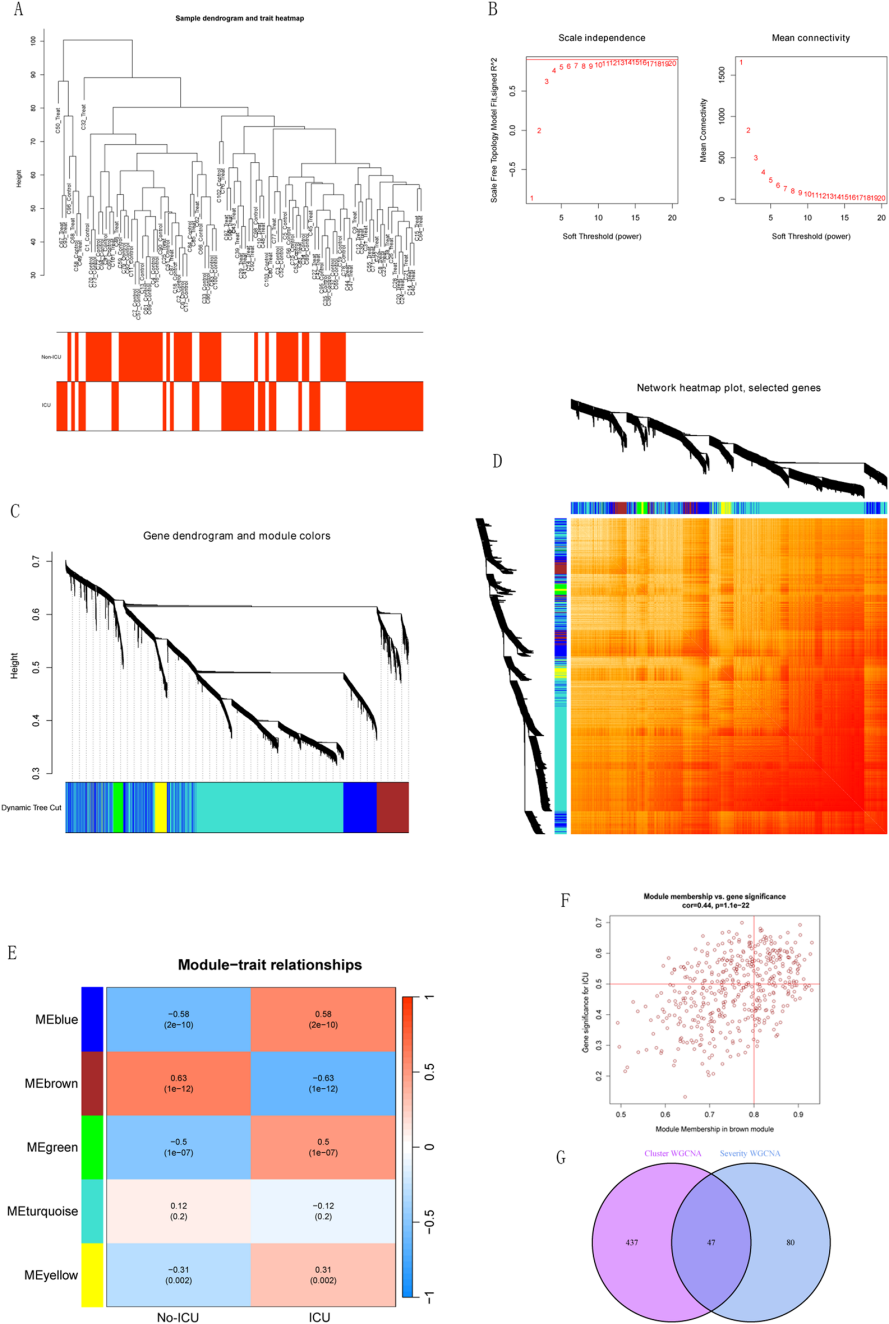
The WGCNA algorithm was used to identify key gene modules closely associated with the COVID-19 Severity respectively. (**A**) The sample dendrogram and feature heat map were drawn based on the Euclidean distance using the average clustering method for hierarchical clustering of samples, with each branch representing a sample, Height in the vertical coordinate being the clustering distance, and the horizontal coordinate being the COVID-19 severity information. (**B**) Soft threshold (power = 13) and scale-free topology fit index (R^2^ = 0.9). (**C**,**D**) Gene hierarchy tree-clustering diagram. The graph indicates different genes horizontally and the uncorrelatedness between genes vertically, the lower the branch, the less uncorrelated the genes within the branch, i.e., the stronger the correlation. (**E**) Heat map showed the relations between the module and treat features 2. The values in the small cells of the graph represent the two-calculated correlation coefficients between the eigenvalues of each trait and each module as well as the corresponding statistically significant p-values. Color corresponds to the size of the correlation; the darker the red, the more positive the correlation; the darker the green, the more negative the correlation. (**F**) Scatter plot between GS and MM in brown. (**G**) Venn diagram demonstrating 47 genes associated with ICU COVID-19.

The WGCNA algorithm was used to identify key gene modules closely associated with the ICD cluster and COVID-19 severity respectively. We constructed a scale-free network using WGCNA (power = 13, R^2^ = 0.9) based on ICD cluster (Fig. 5A). 4868 genes were divided into 5 key modules and the TOM of all module-associated genes was depicted in heat map (Fig. 5B–D). Analysis of module-clinical characteristics relationships showed that the MEblue module (1079 genes) had the highest correlation with Cluster 2 (0.81) and high intra-module gene significance (Fig. 5E). The correlation analysis of MEblue module gene with Cluster 2 is shown in Fig. 5F. Besides, we also constructed a scale-free network using WGCNA (power = 13, R^2^ = 0.9) based on COVID-19 Severity (Fig. 6A). 4868 genes were divided into 5 key modules and the TOM of all module-associated genes was depicted in heat map (Fig. 6B–D). Analysis of module-clinical characteristics relationships showed that the MEbrown module (449 genes) had the highest correlation with Cluster 2 (0.63) and high intra-module gene significance (Fig. 6E). The correlation analysis of MEblue module gene with Cluster 2 is shown in Fig. 6F. The hub genes of each module were screened according to the analysis of the importance of genes and the correlation between genes and modules. 47 intersection genes were obtained through hub genes of MEbrown module and MEblue module mentioned above (Fig. 6G).

### Construction of machine learning models

**Figure 7:**
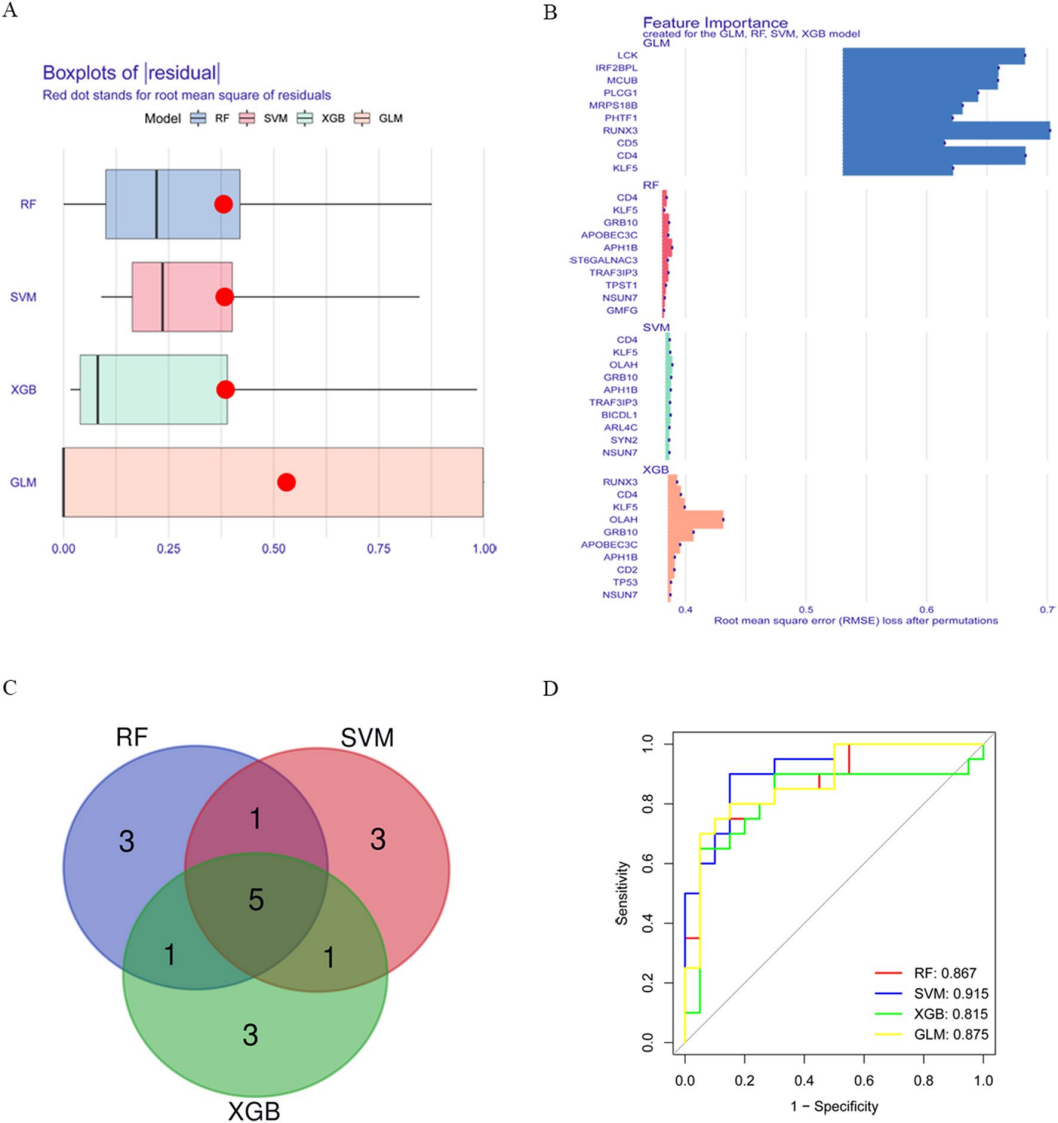
Construction and evaluation of SVM, RF, GLM, and XGB machine models. (**A**) Boxplot showed the residuals of each machine learning model. Red dot represented the root mean square of residuals (RMSE). (**B**) The important features in SVM, RF, GLM, and XGB machine models. (**C**) Venn diagram demonstrating 5 same genes from the intersection of the top 10 significant genes from RF, SVM, and XGB machine learning models. (**D**) ROC analysis of four machine learning models based on 5 same genes.

Four machine learning models, namely RF, SVM, GLM and XGB, were constructed base on the 47 module intersection genes using the fivefold cross-validation to identify specific genes with high diagnostic value for COVID-19 Severity. A “DALEX” package was used to analyze the residual distribution of the above four models. The results showed that the RF, SVM and XGB model all had relatively low residuals (Fig. 7A, Supplementary file 1: Fig. S1). We ranked the top 10 significant genes of each model based on Root mean square error (RMSE) (Fig. 7B). Five same genes were obtained by intersecting the top 10 significant genes of RF, SVM and XGB machine learning models (Fig. 7C). Based on the 5 same genes, the four machine learning models of RF, SVM, GLM and XGB were constructed again and ROC curves were plotted. The AUCs of the four models were, in order, RF: AUC = 0.867; SVM, AUC = 0.915; XGB, AUC = 0.815; and GLM, AUC = 0.875 (Fig. 7D). In conclusion, we concluded that the SVM model was able to better differentiate between the different severities of patients. The SVM model ultimately obtained five significant genes (KLF5, NSUN7, APH1B, GRB10 and CD4), which were used as predictive genes for subsequent analysis.

### Construction and evaluation of the nomogram model

**Figure 8:**
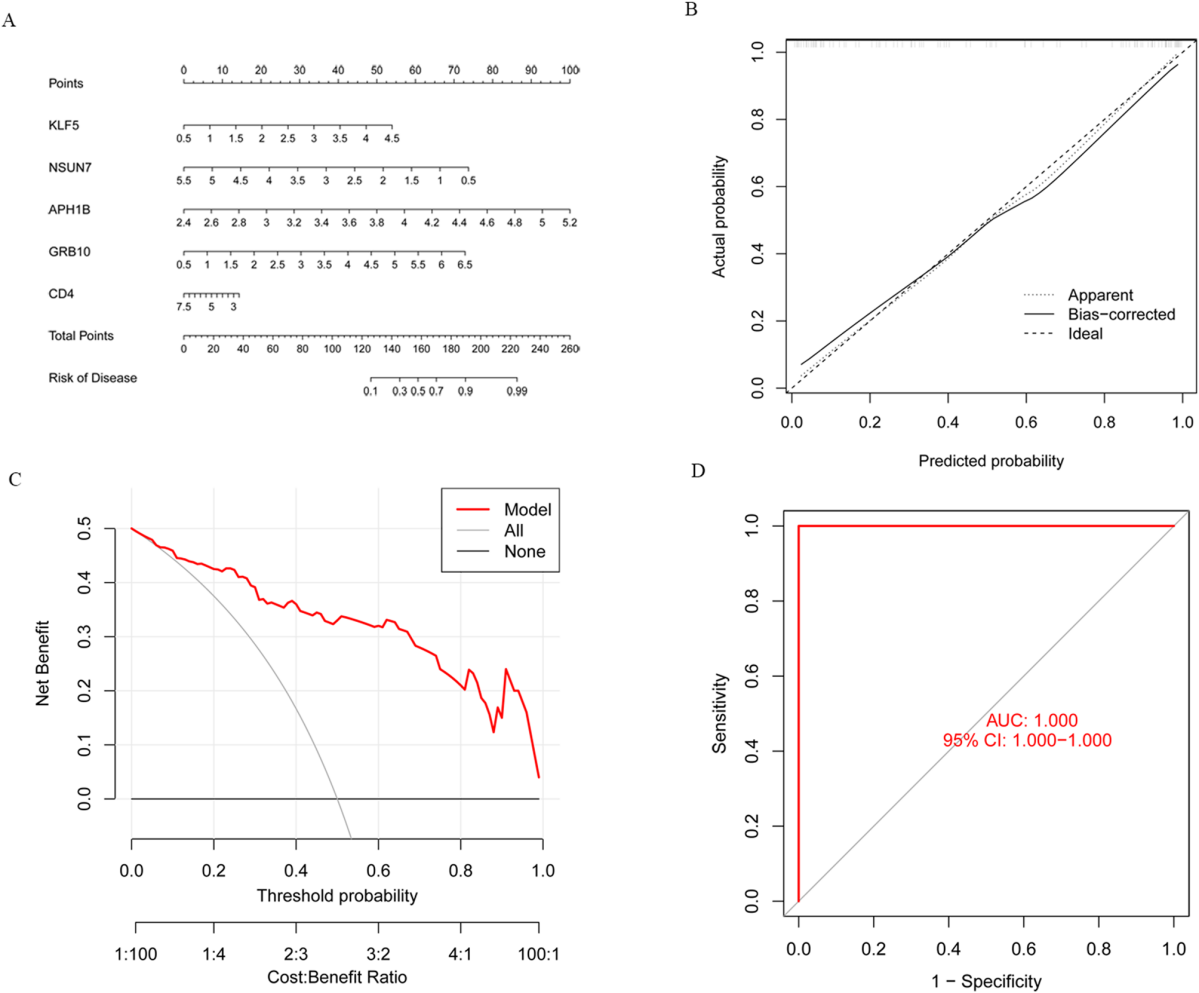
Construction of the nomogram model. (**A**) The ordinary nomogram for the joint prediction of the severe covid-19 based on KLF5, NSUN7, APH1B, GRB10 and CD4. (**B**) Calibration curve for nomogram validation. (**C**) Clinical impact of the nomogram model as assessed by the clinical impact curve. (**D**) ROC analysis for model robustness verification.

To better predict the risk of COVID-19 severity, a nomogram was constructed based on the SVM machine learning model (Fig. 8A). The predictive efficiency of the nomogram model was evaluated using a combination of the calibration curve and DCA, with the results showed that this model has a small error range and high accuracy for clinical treatment decisions (Fig. 8B,C). In addition, an independent external dataset of GSE172114 containing 69 COVID-19 clinical simples (46 critical and 23 non-critical patients) were used to validate the robustness of the SVM machine learning model. The results demonstrated that ROC-AUC of the risk score was 1.000 (95% CI 1.000–1.000), indicating excellent model discrimination (Fig. 8D).

## Discussion

In the past few years, COVID-19 has caused a global pandemic and many deaths. The death of COVID-19 is primarily due to from critically ill patients, especially from ARDS complications caused by SARS-CoV-2. Therefore, early prediction and identification of critically ill patients and active treatment are extremely important to reduce mortality. The current clinical indicators used to predict and identify severe cases of COVID-19 lack sufficient accuracy and have a certain delay. The identification of more appropriate molecular biomarkers is therefore essential to predict severe cases. Studies have shown that a variety of cell death forms are involved in the occurrence and progression of severe COVID-19, such as apoptosis, pyroptosis, NETosis and necroptosis, but the role of immunogenic cell death is rarely reported. In the current study, we attempted to elucidate the relationship between ICD-related genes and the phenotype of COVID-19 severity. Firstly, we extracted the expression matrix of ICD-related genes in the COVID-19 expression dataset and conducted differential expression analysis. Subsequently, we performed consistent cluster analysis based on ICD-related DEGs, Immune infiltration analysis, and used multiple machine learning methods to build models for the identification of COVID-19 severity. Finally, a nomogram for predicting the risk of severe COVID-19 was constructed based on the classification and identification model, and an external dataset was used to validate the model.

Immunogenic cell death, as a specific variant of regulatory cell death, can induce adaptive immune responses against cell death antigens in the host^12^. When cells are stimulated to produce ICDs, dying cells produce new epitopes and release damage associated molecular patterns (DAMPs), which are recognized by pattern recognition receptors (PRRs) and presented to T cells, further activating the adaptive immune response^14^. In severe COVID-19 patients, multiple DAMPs, including HMGB1 and S100 proteins, are significantly increased due to the massive release of inflammatory mediators and the occurrence of cell death, thereby causing the body's immune response disorders and aggravating the progression of COVID-19^21^. In this study, we analyzed the differential expression of ICD-related genes in COVID-19-positive and COVID-19-negative samples and in COVID-19-severe and non-severe samples, respectively. By integration analysis, a total of 9 ICD-related genes (TLR4, HSP90AA1, PIK3CA, BAX, ENTPD1, CD4, CASP1, FOXP3, TNF) were obtained, which were differentially expressed in both of them, suggesting that these ICD-related genes may be closely associated with the occurrence of severe COVID-19 and immune infiltration.

In addition, immune infiltration analysis based on COVID-19 patients showed that ICU patients had low levels of T cell CD8, T cell CD4 memory activation, T cell regulatory (Tregs), NK cell resting, monocytes, macrophages M2 and eosinophils, which was consistent with the studies of Catanzaro M et al.^23^. The decrease in immune cells leads to a delay in the clearance of the virus, which may be the key to the exacerbation of illness in critically ill patients^23,24^. Besides, we found that neutrophils exhibited high infiltration levels in ICU COVID-19 patients. At the same time, we classified COVID-19 patients into two clusters based on ICD-related DEGs in COVID-19-positive and COVID-19-negative samples. Immunoinfiltration analysis based on different clusters also showed that neutrophils exhibited significantly different levels of infiltration in clusters 1 and 2, suggesting that there is some internal relationship between grouping based on different clusters and different severity. Neutrophils, as the main players in innate immunity, are recruited first during viral infection to engulf and clear pathogens. It also produces neutrophil extracellular traps and cytokines that recruit other immune cells to participate in adaptive immune processes while limiting or inactivating the virus^25^. The study of COVID-19 patients by Barnes et al. also showed high infiltration of neutrophils^26^. And Lagunas-Rangel F. A. et al. pointed out that the patients with severe COVID-19 have relatively high neutrophil infiltration. But the correlation and predictive value of neutrophil levels in severe cases remain to be studied^27,28^. Meanwhile, the correlation analysis of ICD-related DEGs and immune cell infiltration in severe COVID-19 showed that TLR4, PIK3CA, FOXP3, ENTPD1, CD4, CASP1 and BAX were significantly correlated with a variety of immune cell infiltration. It is suggested that these immunogenic cell death related genes may be involved in the occurrence and progression of COVID-19 severe disease by inducing the infiltration level of multiple immune cells. There is also a study suggesting an association between COVID-19 and immunogenic cell death and immune cell infiltration, but there is a lack of relevant studies on severe COVID-19^29^. We may be able to give different immunotherapies according to the expression of these ICD-related DEGs and the level of infiltration of immune cells to improve the prognosis of severe COVID-19 patients.

To further accurately screen central genes associated with the severe phenotype of COVID-19, we used WGCNA method to construct gene co-expression networks in COVID-19 samples and COVID-19 severe samples, respectively. 47 intersection genes were obtained through hub genes of Modules most relevant to clinical phenotypes. Four machine learning models were constructed base on the 47 module intersection genes to identify specific genes with high diagnostic value for COVID-19 Severity. We ranked the top 10 significant genes of each model based on Root mean square error (RMSE). Five same genes were then obtained by intersecting the top 10 significant genes of RF, SVM and XGB machine learning models. The SVM model based on 5 same genes (KLF5, NSUN7, APH1B, GRB10 and CD4) was identified as the best prediction model, with the AUC was 0.915. At the same time, its predictive value has been well verified on the verification dataset. A nomogram containing the five genes can better predict the occurrence of severe COVID-19. We are concerned that Zhou et al. have published a study on immunogenic cell death and COVID-19. In the study, they built a predictive model for COVID-19 diagnosis based on ICD-related genes. Similarly, we built a predictive model based on ICD-related genes for the identification of severe COVID-19, which is more clinically significant. Because the diagnosis of COVID-19 is not difficult in clinical practice, compared with biomarkers, etiological diagnosis is the gold standard. On the contrary, how to identify severe COVID-19 patients early and improve the cure rate is the challenge that needs to be solved. However, our predictive model still requires further calibration and validation with large clinical datasets to improve predictive effectivity and accuracy.

KLF5 is a member of the KLF family and plays an important role in various pathophysiological processes such as inflammation, apoptosis and autophagy. Recent studies have shown that it can inhibit viral replication during SARS-CoV-2 infection by controlling sphingolipid metabolism^30^. Jiakai Hou and Janneh A. H respectively pointed out that KLF5 was significantly correlated with the occurrence, development and severity of COVID-19 through multi-omics and clinical observation analysis^31,32^. CD4 gene encodes the CD4 membrane glycoprotein of T lymphocytes, which is mainly expressed in T helper lymphocytes, but also in B cells, macrophages, granulocytes and other cells. CD4 molecules are involved in innate and adaptive immune responses in a variety of ways, including by recognizing antigens displayed by antigen-presenting cells^33,34^. Studies have shown that during viral infection, infection with CD4 + T cells is an effective mechanism for the virus to evade the immune response^35^. The role of CD4 (+) T-cells in SARS-CoV-2 infection has been widely reported. A recent study found that SARS-CoV-2 spike glycoprotein (S) directly binds to CD4 molecules, and then mediates SARS-CoV-2 infection of T helper cells, resulting in CD4 T cell function impairment or even death. In addition, T helper cells infected with SARS-CoV-2 express higher levels of IL-10, which is associated with virus persistence and disease severity^36^. APH1B belongs to the APH-1 family and encodes a multichannel transmembrane protein that is a functional component of the gamma-secretase complex. Studies have shown that the integrity of the gamma-secretase complex plays an important role in inflammation and cell death^37^, but data based on COVID-19 are currently lacking. Grb10 is an adaptor protein, a member of the Grb7/Gr10/Grb14 protein family, and Grb10 is associated with cell proliferation, apoptosis, inflammation and immune regulation^38^. However, whether it regulates cell death and immune response during COVID-19 has not been studied. Sun RNA methyltransferase 7 (NSUN7) belongs to the methyltransferase superfamily, which is the dominant RNA m5C modifying enzyme in eukaryotes. A study has suggested that the mean precursor strength of plasma protein polypeptides, such as NSUN7, is increased in patients with sepsis^39^. Another study reported that NSUN7 may be associated with an excessive inflammatory response in sepsis^40^. In addition, NSUN7 has been reported to be closely associated with immune and inflammatory responses and may be a biomarker for the pathogenesis of neonatal sepsis^41^. After SARS-CoV-2 infection, some severe patients will develop sepsis. However, whether NSUN7 is associated with sepsis caused by SARS-CoV-2 remains unclear. The aforementioned studies involving these characteristic genes show that our screening results are reliable to a certain extent, but the biological functions of some genes and their roles in the occurrence and progression of COVID-19 severe diseases still need further experimental exploration. In the future, we can explore their potential mechanism of action in severe COVID-19 patients through molecular biology experiments, and provide directions for subsequent targeted and immunotherapy for severe COVID-19 patients.

In conclusion, we systematically analyzed the role of ICD-related genes in COVID-19. The differential expression of ICD-related genes and immune infiltration in COVID-19 samples and healthy control samples, as well in ICU samples and Non-ICU samples were respectively explored. A prediction model for severe COVID-19 was constructed based on ICD-related genes via consensus cluster analysis, weighted gene co-expression network analysis and multiple machine learning algorithms. This study may provide a valuable direction for the study of the pathogenesis, progression and immunotherapy of COVID-19, and at the same time provide help for the early identification of severe COVID-19.

## Materials and methods

### Datasets and differentially expressed genes analysis

**Figure 9:**
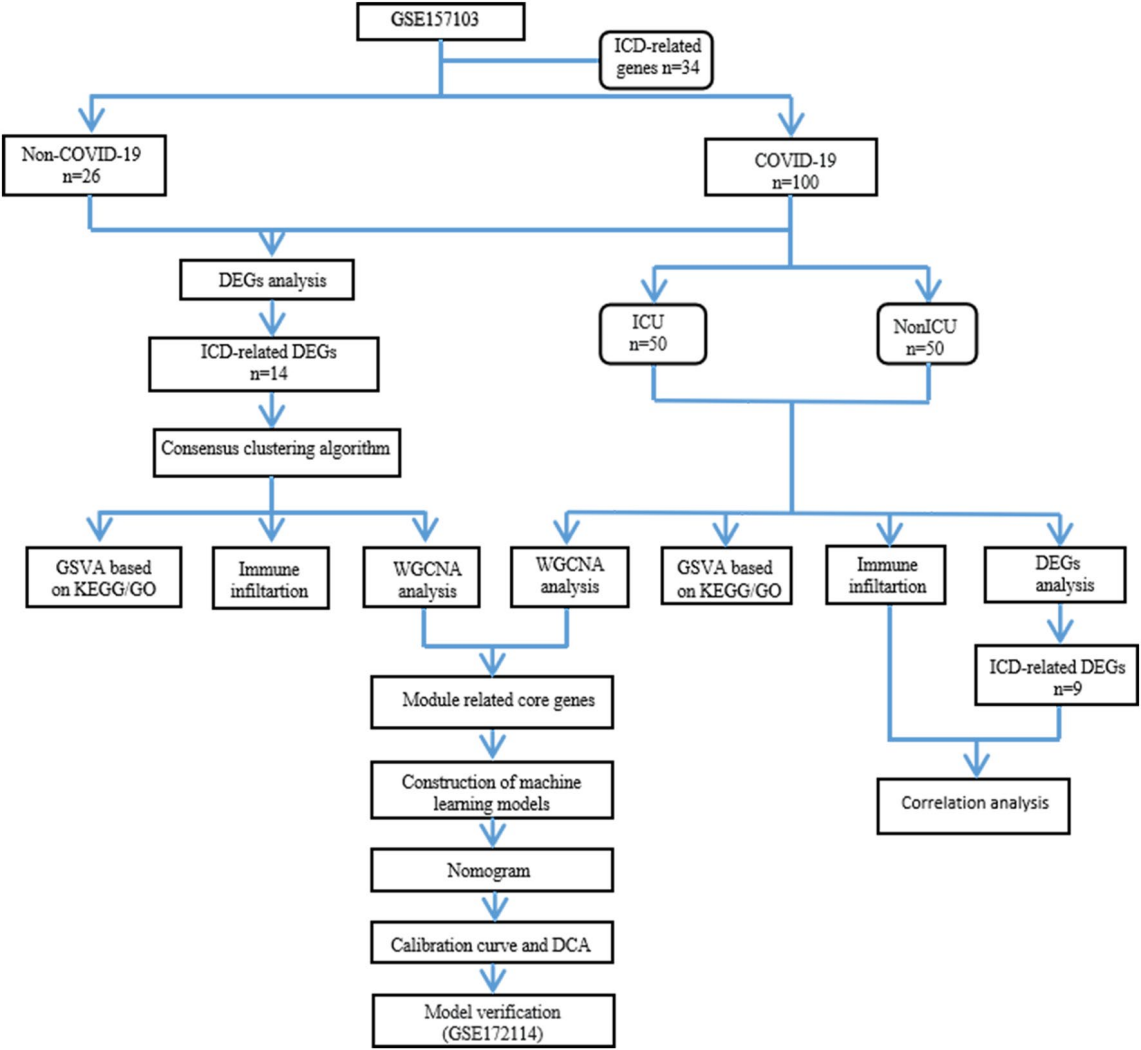
Flowchart of the whole study.

Gene expression sequencing datasets of COVID-19-related peripheral blood samples were obtained via the gene expression omnibus (GEO) database. A large-scale sequencing dataset GSE157103^42^ was used as a training set, which provides transcriptomic data from whole blood obtained from a population of COVID-19 patients ( n = 100, including 50 ICU and 50 NonICU patients) and Non-COVID-19 patients (n = 26). The sequencing dataset GSE172114^43^, containing 46 critical COVID-19 patients and 23 non-critical COVID-19 patients, was used as an external validation set. Both the training set and validation set are from GPL24676 Illumina NovaSeq 6000 (Homo sapiens). The differentially expressed genes (DEGs) were identified by using “Limma” package in version 4.1.2 R. A p-value < 0.05 was considered to be statistically significant. A flowchart representing the overall concepts and procedures employed in this study is shown in Fig. 9.

### Immune cell infiltration profile

The relative content of 22 immune cells in each sample was estimated through the CIBERSORT package^44^. Then, we used the Wilcoxon test to compare the proportion of immune cells between different groups.

### Consensus clustering of COVID-19 patients

Based on ICD-related DEGs, we used “ConsensusClusterPlus” package^45^ to perform unsupervised cluster analysis by classifying 100 COVID-19 samples into different clusters using the k-means algorithm. The optimal cluster number (k) was determined based on the consensus matrix, cumulative distribution function (CDF) curve, and consistent cluster score.

### Pathway enrichment analysis in different severity subtypes and ICD clusters

The “c2.cp.kegg.symbols” file was downloaded from the MSigDB database on the GSEA website (https://www.gsea-msigdb.org/gsea/downloads.jsp). Then, we used the “Gene set variation analysis (GSVA)”^46^ and “Limma” R packages to analyze the altered pathways in different COVID-19 severity subtypes and ICD clusters respectively.

### WGCNA analysis

In the ICD cluster and COVID-19 Severity datasets, we selected the top 25% genes with the largest fluctuations respectively for WGCNA analysis^47^. Firstly, we constructed the gene co-expression matrix by calculating Pearson correlation coefficient between gene pairs using the R WGCNA package^48^. According to the scale-free network principle, a scale-free co-expression network was constructed by the best soft threshold, and the adjacency matrix was converted into topological overlap matrix (TOM). Based on a hierarchical clustering tree algorithm, we used a TOM dissimilarity measure (1-TOM) to identify gene modules, with at least 100 genes per module. The hierarchical clustering method was used to construct the dendrogram, and the correlation between module feature genes and disease phenotypes was calculated. The module with the highest correlation coefficient and the lowest P-value was defined as the disease feature module. Gene significance (GS) and module membership (MM) were calculated for the genes of each module respectively, and the core genes of the module were screened according to the criteria of GS > 0.5 and MM > 0.8^47^.

### Constructing predictive models based on multiple machine learning methods

Four machine learning methods of random forest model (RF)^49,50^, support vector machine model (SVM)^51^, generalised linear model (GLM)^52^, and eXtreme Gradient Boosting (XGB)^53^were performed to accurately construct predictive models based on different severity subtypes by using the ‘caret’ package in R. The 100 COVID-19 samples were randomly divided into training set (60%, N = 60) and validation set (40%, N = 40). To produce an optimal model, we performed hyperparameter tuning for each machine learning method using fivefold cross-validation. Residual distribution and feature importance of the four machine learning models were visualized using the “DALEX” package in R. The area under the receiver operating characteristic (ROC) curve (Area Under Curve, AUC) was executed and visualized using the “pROC” package. The best machine learning model was determined according to residual distribution and AUC.

### Construction and validation of the nomogram model

The five predictor genes obtained based on the best machine learning model were used to construct a nomogram in “rms” R package^54^ to assess the predictive probability for the severity of COVID-19. Each predictor was assigned a score based on the expression value of the gene, and the 'total score' of the sum of all predictors scores corresponded to the probability of a critical disease risks. The predictive power of the nomogram was assessed by using the calibration curve and DCA.

### Statistical analysis

The bioinformatics analyses and R packages used in this study were all performed using R software (version 4.2.1). The two-tailed unpaired Student's t test was used to compare the normal distribution data between the two groups. The non-normally distributed data were compared by Wilcoxon test. p < 0.05 was considered statistically significant (*p < 0.05, **p < 0.01, ***p < 0.001, ****p < 0.0001).

### Supplementary Information

Supplementary Figure S1.

Supplementary Information 1.

Supplementary Information 2.

## Data Availability

The datasets used in the present study are from the publicly available datasets GSE157103 and GSE172114, which can be downloaded from the GEO Dataset (https://www.ncbi.nlm.nih.gov/gds). The process and method of analysis have been shown in the article, and further data will be made available from the corresponding author on reasonable request.
