# Supplementary figures and images for: Comprehensive analysis of immunogenic cell death-related gene and construction of prediction model based on WGCNA and multiple machine learning in severe COVID-19
Source: Sci Rep. 2024 Apr 11;14:8450. doi: 10.1038/s41598-024-59117-0 (PMC11006847; doi:10.1038/s41598-024-59117-0)

# Reverse cumulative distribution of |residual|

Model — RF — SVM — XGB — GLM

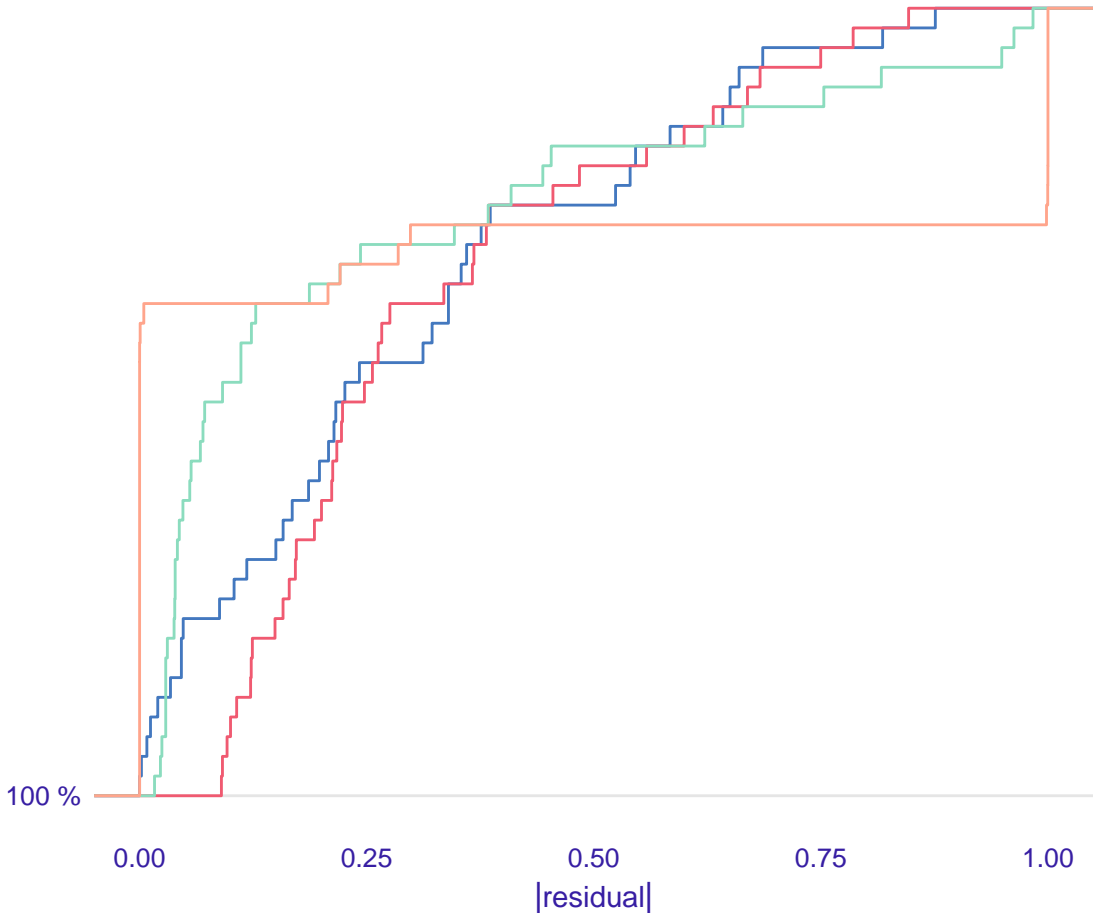

Supplement: Supplementary file 1 — Supplementary Figure S1. [file 41598_2024_59117_MOESM1_ESM.pdf]
